# Supplementary material for: The baseline immunological and hygienic status of pigs impact disease severity of African swine fever
Source: PLoS Pathog. 2022 Aug 25;18(8):e1010522. doi: 10.1371/journal.ppat.1010522 (PMC9409533; doi:10.1371/journal.ppat.1010522)
Supplement: S1 Fig — (A) Hematocrit, hemoglobin and mean corpuscular hemoglobin concentration (MCHC) counts in uninfected pigs. (B) Immunophenotyping of blood leukocytes (CD45+). Percentage of each subset was determined by flow cytometry gating. (C) T cell subsets were gated from CD3+ T cells. (A-C) Each point represents the value for a single pig, horizontal lines and boxes represent the mean and range. Data are from 2 independent experiments (n = 15 per SPF or farm groups) and were analyzed using unpaired t test; * p<0.05; ** p<0.01; *** p<0.001. (PDF) [file ppat.1010522.s001.pdf]

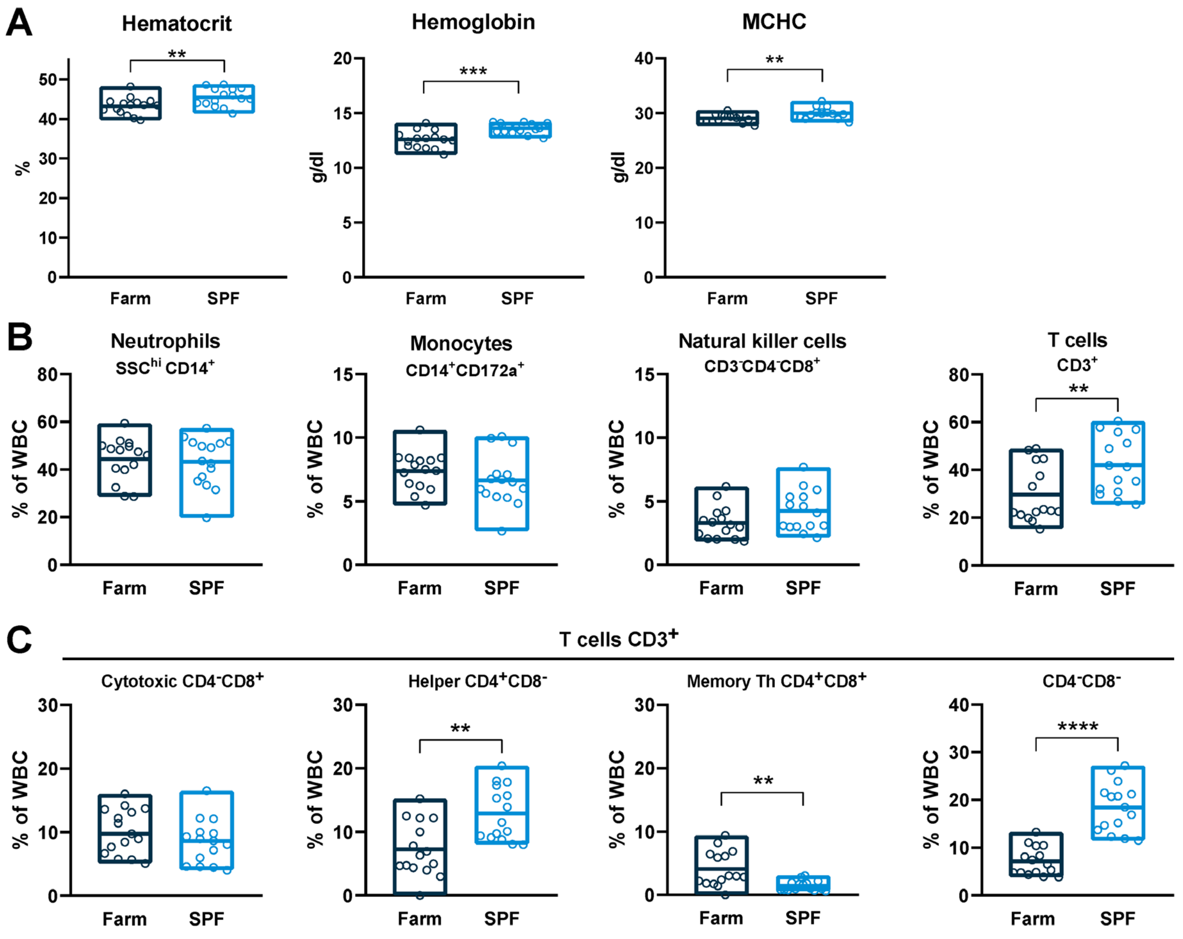

S1 Fig. Basal hematologic profiles of SPF and farm pigs. (A) Hematocrit, hemoglobin and mean corpuscular hemoglobin concentration (MCHC) counts in uninfected pigs. (B) Immunophenotyping of blood leukocytes ($CD45^+$). Percentage of each subset was determined by flow cytometry gating. (C) T cell subsets were gated from $CD3^+$ T cells. (A-C) Each point represents the value for a single pig, horizontal lines and boxes represent the mean and range. Data are from 2 independent experiments (n=15 per SPF or farm groups) and were analyzed using unpaired t test; * p<0.05; ** p<0.01; *** p<0.001.
