# Supplementary material for: The baseline immunological and hygienic status of pigs impact disease severity of African swine fever
Source: PLoS Pathog. 2022 Aug 25;18(8):e1010522. doi: 10.1371/journal.ppat.1010522 (PMC9409533; doi:10.1371/journal.ppat.1010522)
Supplement: S2 Fig — (A, B) Representative flow cytometry dot plots of single cells with gates for total leukocytes (WBC, CD45+), neutrophils (CD45+SSChiCD14+) and monocytes (CD45+SSClowCD14+CD172a+) of farm (A) and SPF (B) pigs measured at baseline. (C, D) Representative flow cytometry dot plots of leukocytes (WBC, CD45+) with gates for T cell subsets (CD3+) based on CD4 and CD8 markers and NK cells (CD3-CD8+) in farm (C) and SPF (D) pigs measured at baseline. (PDF) [file ppat.1010522.s002.pdf]

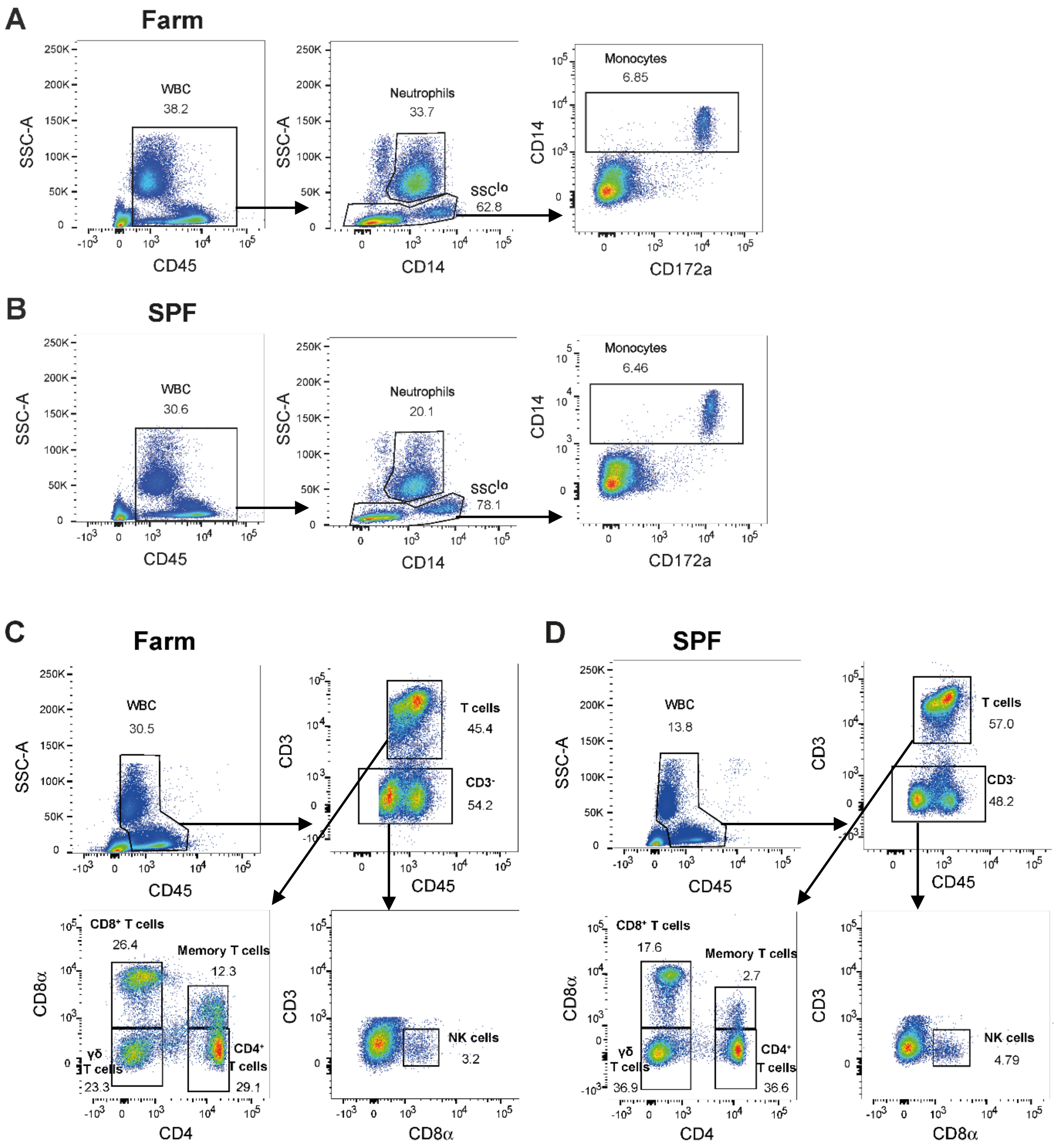

S2 Fig. Flow cytometry gating strategies for blood leukocyte immunophenotyping. (A, B) Representative flow cytometry dot plots of single cells with gates for total leukocytes (WBC, $CD45^{+}$), neutrophils ($CD45^{+}SSC^{hi}CD14^{+}$) and monocytes ($CD45^{+}SSC^{low}CD14^{+}CD172a^{+}$) of farm (A) and SPF (B) pigs measured at baseline. (C,D) Representative flow cytometry dot plots of leukocytes (WBC, $CD45^{+}$) with gates for T cell subsets ($CD3^{+}$) based on CD4 and CD8 markers and NK cells ($CD3^{-}CD8^{+}$) in farm (C) and SPF (D) pigs measured at baseline
