# Supplementary material for: The baseline immunological and hygienic status of pigs impact disease severity of African swine fever
Source: PLoS Pathog. 2022 Aug 25;18(8):e1010522. doi: 10.1371/journal.ppat.1010522 (PMC9409533; doi:10.1371/journal.ppat.1010522)
Supplement: S3 Fig — Animals from 2 independent experiments are included (farm group n = 15, SPF group n = 11). (PDF) [file ppat.1010522.s003.pdf]

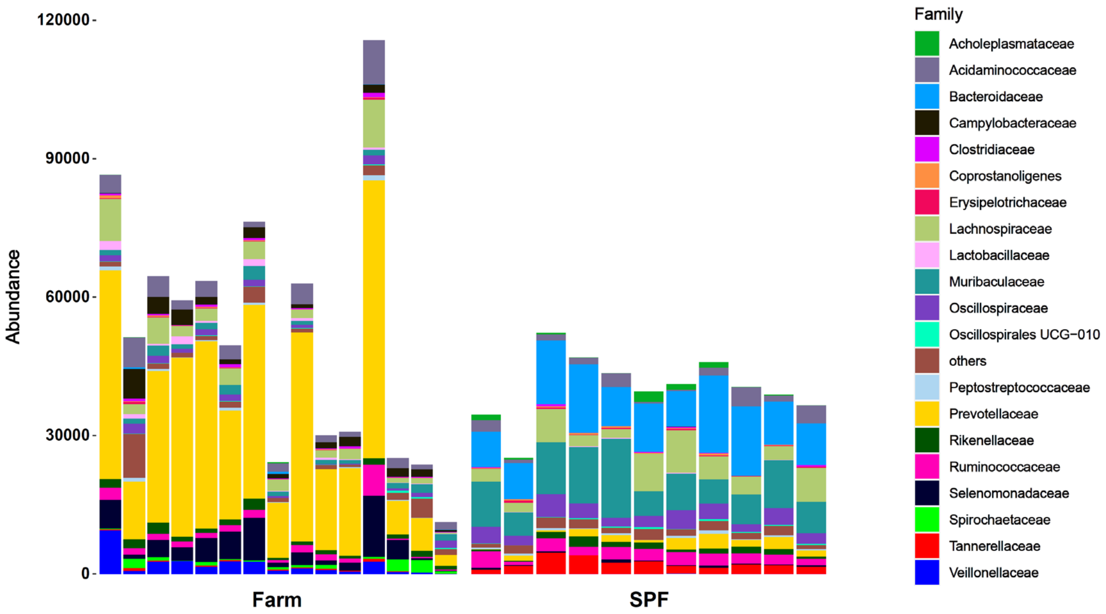

S3 Fig. Abundance of the most frequent bacterial families present in fecal microbiota of farm and SPF pigs at steady state. Animals from 2 independent experiments are included (farm group n=15, SPF group n=11).
