# Supplementary material for: The baseline immunological and hygienic status of pigs impact disease severity of African swine fever
Source: PLoS Pathog. 2022 Aug 25;18(8):e1010522. doi: 10.1371/journal.ppat.1010522 (PMC9409533; doi:10.1371/journal.ppat.1010522)
Supplement: S4 Fig — SPF and farm pigs were inoculated intramuscularly with ASFV Armenia 2008. Blood samples were taken 1 day before infection and 1, 2, 4, 5, and 7 dpi. (A) Hematocrit, hemoglobin and mean corpuscular hemoglobin concentration (MCHC) counts in blood at indicated dpi. (B) Percentage of leukocyte (CD45+). subsets in blood determined by flow cytometry (S2 Fig). Data points represent values for individual pigs, lines indicate mean of each group. Data are from a single experiment (n = 6 pigs/group, except 7 dpi, where n = 3 for SPF group). Differences between SPF and farm groups were analyzed by unpaired t test at each dpi with Holm-Sidak’s correction for multiple comparisons. * p<0.05; ** p<0.01; *** p<0.001. (C) T cell subsets gated on CD45+CD3+. (PDF) [file ppat.1010522.s004.pdf]

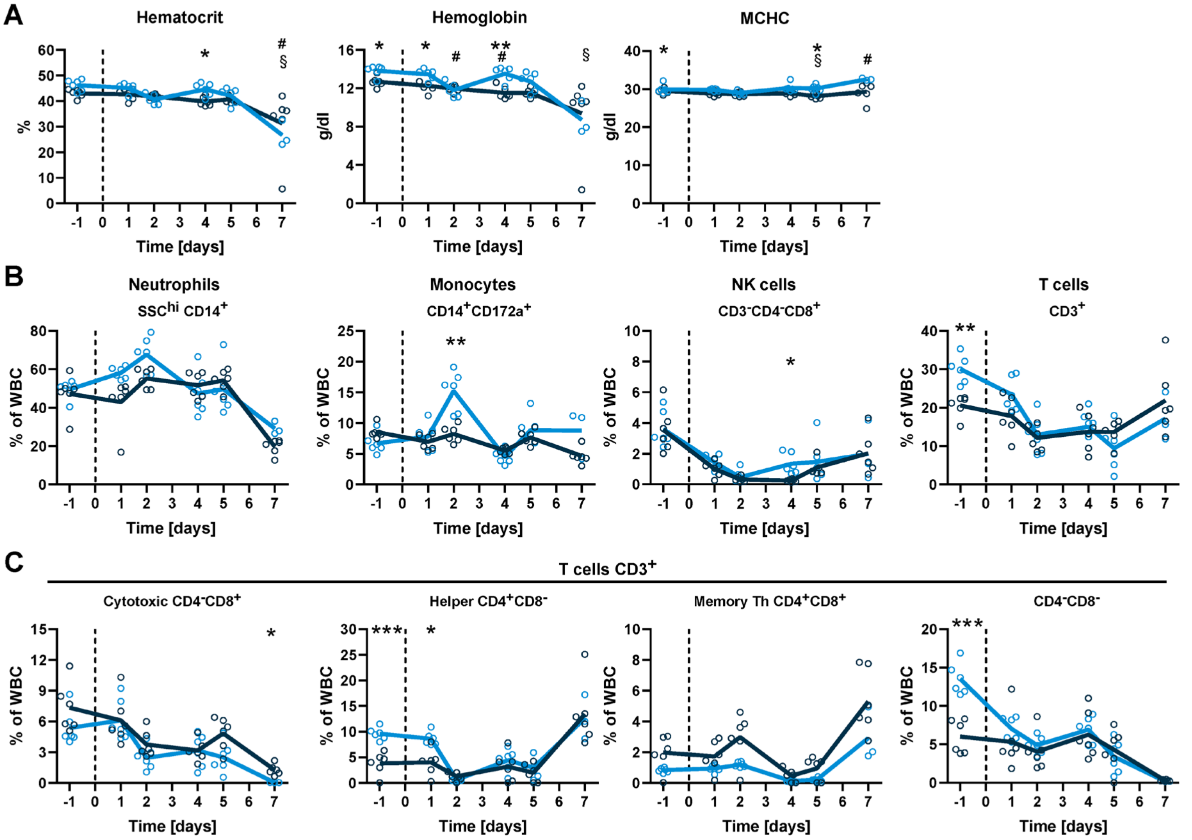

S4 Fig. Blood cell profiles after infection with virulent ASFV strain Armenia 2008. SPF and farm pigs were inoculated intramuscularly with ASFV Armenia 2008. Blood samples were taken 1 day before infection and 1, 2, 4, 5, and 7 dpi. (A) Hematocrit, hemoglobin and mean corpuscular hemoglobin concentration (MCHC) counts in blood at indicated dpi. (B) Percentage of leukocyte ($CD45^+$) subsets in blood determined by flow cytometry (Suppl. Fig. S2). Data points represent values for individual pigs, lines indicate mean of each group. Data are from a single experiment (n=6 pigs/group, except 7 dpi, where n=3 for SPF group). Differences between SPF and farm groups were analyzed by unpaired t test at each dpi with Holm-Sidak's correction for multiple comparisons. * $p<0.05$; ** $p<0.01$; *** $p<0.001$. (C) T cell subsets gated on $CD45^+CD3^+$.
