# Supplementary material for: The baseline immunological and hygienic status of pigs impact disease severity of African swine fever
Source: PLoS Pathog. 2022 Aug 25;18(8):e1010522. doi: 10.1371/journal.ppat.1010522 (PMC9409533; doi:10.1371/journal.ppat.1010522)
Supplement: S5 Fig — SPF and farm pigs were inoculated intramuscularly with ASFV Estonia 2014. Blood samples were taken 1 day before infection and 1, 2, 4, 5, 7, 11, 14 and 26 dpi. (A) Virus load in organs was measured by qPCR on the day of euthanasia (5 dpi grey circles crossed (SPF n = 1), 6 dpi, grey circles (SPF n = 2), 7 dpi, empty circles (SPF n = 3, farm n = 6)). SM LN, submandibular lymph node; GH LN, gastrohepatic LN; BM, bone marrow; Saliv. gl., salivary gland. (B) Hematocrit, hemoglobin and mean corpuscular hemoglobin concentration (MCHC) values in blood. (C) Percentage of leukocyte (CD45+). subsets in blood determined by flow cytometry (S2 Fig). (D) T cell subsets gated from CD3+ T cells. Data are from a single experiment (n = 6 pigs/group, except from 11 dpi (n = 5) and from 15 dpi (n = 3) for farm group). (B-D) Differences between SPF and farm groups were analyzed by unpaired t test at each dpi with Holm-Sidak’s correction for multiple comparisons. * p<0.05; ** p<0.01; *** p<0.001; **** p<0.0001. (PDF) [file ppat.1010522.s005.pdf]

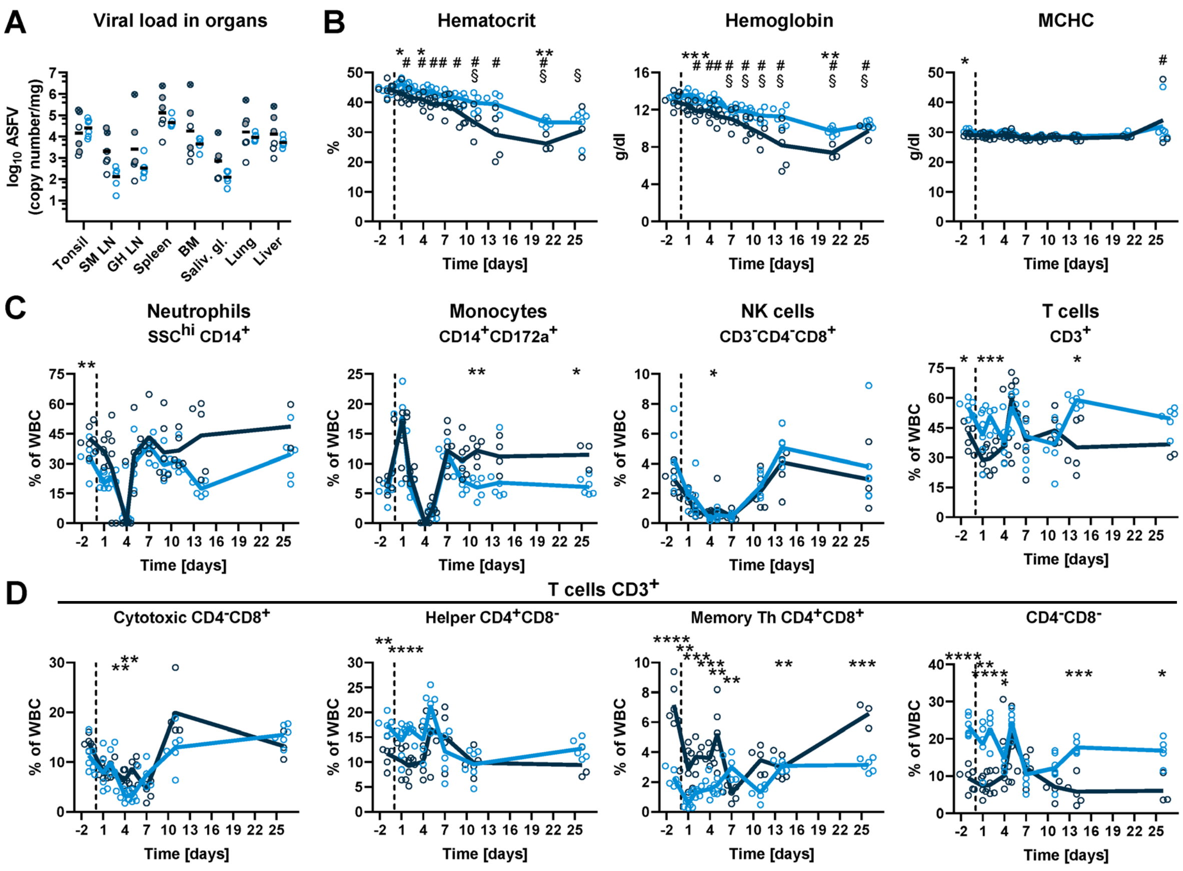

S5 Fig. Viral load and blood cell profiles after infection with attenuated ASFV strain Estonia 2014. SPF and farm pigs were inoculated intramuscularly with ASFV Estonia 2014. Blood samples were taken 1 day before infection and 1, 2, 4, 5, 7, 11, 14 and 26 dpi. (A) Virus load in organs was measured by qPCR on the day of euthanasia (5 dpi grey circles crossed (SPF n=1), 6 dpi, grey circles (SPF n=2), 7 dpi, empty circles (SPF n=3, farm n=6)). SM LN, submandibular lymph node; GH LN, gastrohepatic LN; BM, bone marrow; Saliv. gl., salivary gland. (B) Hematocrit, hemoglobin and mean corpuscular hemoglobin concentration (MCHC) values in blood. (C) Percentage of leukocyte ($CD45^+$) subsets in blood determined by flow cytometry (Suppl. Fig. S2). (D) T cell subsets gated from $CD3^+$ T cells. Data are from a single experiment (n=6 pigs/group, except from 11 dpi (n=5) and from 15 dpi (n=3) for farm group). (B-D) Differences between SPF and farm groups were analyzed by unpaired t test at each dpi with Holm-Sidak's correction for multiple comparisons. * $p<0.05$; ** $p<0.01$; *** $p<0.001$; **** $p<0.0001$**.**
