# Supplementary material for: The baseline immunological and hygienic status of pigs impact disease severity of African swine fever
Source: PLoS Pathog. 2022 Aug 25;18(8):e1010522. doi: 10.1371/journal.ppat.1010522 (PMC9409533; doi:10.1371/journal.ppat.1010522)
Supplement: S1 Table — (DOCX) [file ppat.1010522.s006.docx]

| **Viruses** |
| --- |
| Transmissible gastroenteritis virus (TGEV) |
| Porcine epidemic diarrhea virus (PEDV) |
| Porcine respiratory coronavirus (PRCV) |
| Classical swine fever virus (CSFV) |
| African swine fever virus (ASFV) |
| Porcine reproductive and respiratory syndrome virus (PRRSV) |
| Foot-and-mouth disease virus (FMDV) |
| Porcine reproductive and respiratory syndrome virus (SVD) |
| Suid herpesvirus (SuHV1) |
| Porcine parvovirus (PPV) |
| Swine influenza A virus (SIV A) |
| Porcine circovirus type 2 (PCV-2) |
| **Bacteria** |
| *Actinobacillus pleuropneumoniae* |
| *Mycoplasma hyopneumoniae* |
| *Streptococcus suis* |
| *Haemophilus parasuis* |
| *Erysipelothrix rhusiopathiae* |
| *Bordetella bronchiseptica* |
| *Pasteurella multocida* |
| *Lawsonia intracellularis* |
| *Chlamydia sp.* |
| *Brucella sp.* |
| *Leptospira sp.* |

**S1 Table. List of pathogens excluded from SPF pigs**.
