# Supplementary material for: The baseline immunological and hygienic status of pigs impact disease severity of African swine fever
Source: PLoS Pathog. 2022 Aug 25;18(8):e1010522. doi: 10.1371/journal.ppat.1010522 (PMC9409533; doi:10.1371/journal.ppat.1010522)
Supplement: Attachment — Submitted filename: ResponseToReviewers_final.docx [file ppat.1010522.s007.docx]

**Point-by-point response to reviewers**

The reviewers found the manuscript well written and that it addressed important issues directed at understanding susceptibility of pigs, of defined immune and health status, to virulent and an attenuated African swine fever virus (ASFV) isolate. Detailed results are presented which significantly extend previous studies regarding the immune status, microbiome and blood transcriptional responses in infected pigs. These results would be of benefit to the scientific community and more specifically to other researchers interested in pathogenesis of ASFV including evaluating of candidate vaccines. as well as more broadly.

Reviewers 1 and 2 felt that the data presented did not adequately support the conclusions drawn. Experiments are lacking to demonstrate that the measured immune responses in the SPF pigs and the farm pigs are the cause of the reported differences in response. One reviewer suggests additional experiments to support the conclusions. These reviewers also comment that previous publications relevant to your manuscript had not been cited and/or that differences between your results and previous results had not been adequately discussed.
The third reviewer has concerns about the gating strategy used in the FACS experiments. These concerns may be possible to address by further analysis of the data or considered in discussion. The reviewer also questioned the descriptions of use of the anti-CD45 antibody.

Any revision of your manuscript should address the points made by the reviewers.

**Response**

We thank the reviewers and the editors for the careful feedback and the opportunity to submit a revised version of our manuscript. All questions are addressed point by point below.

Reviewer's Responses to Questions

**Part I - Summary**
Please use this section to discuss strengths/weaknesses of study, novelty/significance, general execution and scholarship.

Reviewer #1:

African swine fever (ASF) has become without any doubt, the number one threat for the swine industry worldwide.
ASF research has been traditionally neglected, considering it an “exotic” problem and today, being a global problem, there is not enough knowledge to rapidly counteract its progression.

In this aspect, the article here presented tackles a very important issue, such as it is, understanding the immunological mechanisms involved in susceptibility to ASFV.

On this regard, my first concern is that authors have ignored seminal findings describing that specific pathogen-free (SPF) pigs are much more susceptible to attenuated ASFV strains than conventional pigs (King et al., Vaccine. 2011 Jun 20; 29(28): 4593–4600; Lacasta et al. J Virol. 2014 Nov;88(22):13322-32.); exactly the opposite results here presented. Not mentioning these works in the introduction, from the very beginning, is somehow surprising but not citing them in the discussion is even more difficult to explain. Work performed by two research groups showed higher susceptibility of SPF pigs to two different attenuated strains of ASFV (OURT 88/3 and E75CV1), both of them form the Genotype I isolated in the Iberian peninsula during the endemic period (1957-1997).

I strongly recommend revising the entire manuscript taking into account this reality and the fact that SPF and conventional pigs used in these studies are most probably from different origins than the ones used in the present work.

I encourage the authors to unmask the differences observed between both set of results since the conclusions raised will be without any doubt extremely useful for the scientific community and for the future development of prevention and control of ASF. Without this information the message sent should be only circumscribed to the experimental animals used in the study, resting novelty and relevance to the study.

In fact, the conclusions raised will have to be softened.

**Response**

We agree with the reviewer that we should discuss our results more broadly and compare them with the previous studies. We have thus modified the manuscript to include the citations for the two seminal studies highlighted by the reviewer. These studies principally investigated adaptive immune responses to a challenge with a virulent genotype I strain after DNA vaccination or after immunization with attenuated strains. These studies did not directly aim to compare SPF and farm pigs side by side with the same challenge and the SPF/farm groups were not necessarily always well matched for genetic background, age, and weight. Nevertheless, they provide important insight that we have now discussed. To highlight overall differences in enhanced/reduced susceptibility to attenuated strains in SPF vs farm pigs, we have expanded the manuscript discussion, the description of our SPF pig facility in methods and S1 Table, as follows:

Discussion Lines 253-269 : “Specific-pathogen free (SPF) status of domestic pigs was previously associated with an acute, lethal disease course upon infection with the cell-culture passaged E75CV1 genotype I strain of ASFV that was shown to be attenuated in commercial domestic pigs (Lacasta et al, 2014). Similarly, successive inoculation with the OURT88/3 and OURT88/1 attenuated genotype I strains caused clinical symptoms and death more frequently in SPF pigs than in farm pigs (King et al, 2011). The results of these two studies appear to be at odds with our findings that SPF pigs are less susceptible to severe disease caused by the genotype II Estonia 2014 ASFV strain. The different results may be due to intrinsic differences of genotype I and II strains, or more specifically, caused by different gene deletions and associated mechanisms of attenuation. Moreover, differences in clinical course and immune responses of SPF pigs after ASFV inoculation are likely caused by specific host immune and environmental factors such as the microbiota. SPF status is principally the exclusion of known porcine pathogens (defined in S1 Table for our study) and is not uniform in all facilities, especially regarding baseline immune status and bacterial commensals. We found that the microbiota and baseline immune status are very stable over time in SPF pigs from the IVI facilities and in pigs obtained from the same commercial farm at several months interval. Specific immune cell phenotype, blood transcriptomic profile and microbiota identified here in each group may thus serve as a reference for future studies to better understand factors associated with disease severity and ultimately protective adaptive immune responses.”

Methods Lines 317-326:

“The pigs from the IVI SPF facility are not vaccinated and are negative for all porcine viral and bacterial pathogens listed in S1 Table. Access to the SPF facility is restricted to animal caretakers and includes showering airlock at entrance. The SPF facility is supplied with HEPA-filtered air at positive pressure and pigs are provided irradiated pig pellet diet and autoclaved hay and straw. No animal has been introduced in the facility since the initial colonization in 1993 and no disease clinical signs (diarrhea, abortion, respiratory symptoms) were observed during this period. The genetic pool is maintained at a similar status as current Swiss/European Large White pig farms via artificial insemination using semen from SUISAG (Sempach, Switzerland). The commercial farm pigs were from a high standard fattening pig farm and were vaccinated against porcine circovirus 2, *Escherichia coli*, and *Lawsonia intracellularis*.”

Reviewer #2:

Radulovic et al. present a detailed, well written paper describing the immune status, microbiome and transcriptional response in two groups of domestic pigs after infection with two different isolates of African swine fever virus with differing virulence. As the authors describe African swine fever is a serious threat to animal welfare and food security and the disease/immune status as well as the genetic background of animals is something that has not been widely explored. Such data will be important to help inform decisions around the potential deployment of live attenuated vaccines in the field.
The strengths of the study are the robust and detailed datasets generated, however there are a number of issues which I don't think can be realistically addressed.
1) The data is descriptive in nature, there are no experiments to demonstrate that the measured immune status/microbiome of the SPF pigs and the domestic pigs are the cause of the reported disease progression.

**Response:**

We thank the reviewer for the positive comments. Our detailed findings strongly support the notion that the immune and microbiota status impact disease severity of ASF by performing side-by-side analysis of two groups of animals with a defined background. The results are a strong basis for further mechanistic studies investigating the host side of the disease, and are complementary to studies focused on the intrinsic impact of specific ASFV genes on pathogenesis and immune responses. Our “robust and detailed datasets” set the stage for further mechanistic studies by providing strong leads that can be further explored.

2) The authors results are at odds with existing data with the Estonia 2014 strain. In two separate experiments farm pigs exhibit reduced signs of disease after infection with Estonia 2014 when compared to minipigs/wild boar that were raised in an institutional facility (Zani, 2018; Sehl, 2020). One difference between these previous experiments and the author's were the challenge route (oronasal versus intramuscular).

**Response:**

As discussed in the manuscript, we do not find our results particularly at odds with other in vivo studies of Estonia 2014 but rather provide complementary information to those obtained in domestic pigs, minipigs, and wild boars. In our hands, commercial farm pigs also showed moderate severity as shown in other studies (Zani 2018; Sehl, 2020; Schäfer 2021). It is likely that there will be varying severity depending on the general health and immune status of any commercial or institutional animal holding. By better defining the basal status prior to infection,

In a recent independent animal experiment using SPF and farm pigs from the same sources, groups of SPF and farm pigs were inoculated with the ASFV Estonia 2014 strain via the oro-nasal route. As shown in the figure below, we replicated our findings showing prolonged clinical signs in SPF (light blue) and farm (dark blue) pigs also via the oro-nasal route. We observed that in the oronasal infection the onset of viremia and clinical signs started 2 days later than in the intramuscular infection. These data are part of a follow-up study investigating whether surviving SPF and farm pigs would be protected against a challenge with a virulent Armenia 2008 strain.

3) Although not explored to the same depth as in the present manuscript differences between SPF and farm animals in the context of African swine fever virus infection have been previously reported (Lacasta, 2014).

**Response:**

As discussed above in our response to reviewer 1, we have added additional discussion of previous studies using SPF pigs in the context of ASFV infection. We thank the reviewer for the opportunity to discuss our findings in the context of previous studies.

Discussion Lines 253-269 :

“Specific-pathogen free (SPF) status of domestic pigs was previously associated with an acute, lethal disease course upon infection with the cell-culture passaged E75CV1 genotype I strain of ASFV that was shown to be attenuated in commercial domestic pigs (Lacasta et al, 2014). Similarly, successive inoculation with the OURT88/3 and OURT88/1 attenuated genotype I strains caused clinical symptoms and death more frequently in SPF pigs than in farm pigs (King et al, 2011). The results of these two studies appear to be at odds with our findings that SPF pigs are less susceptible to severe disease caused by the genotype II Estonia 2014 ASFV strain. The different results may be due to intrinsic differences of genotype I and II strains, or more specifically, caused by different gene deletions and associated mechanisms of attenuation. Moreover, differences in clinical course and immune responses of SPF pigs after ASFV inoculation are likely caused by specific host immune and environmental factors such as the microbiota. SPF status is principally the exclusion of known porcine pathogens (defined in S1 Table for our study) and is not uniform in all facilities, especially regarding baseline immune status and bacterial commensals. We found that the microbiota and baseline immune status are very stable over time in SPF pigs from the IVI facilities and in pigs obtained from the same commercial farm at several months interval. Specific immune cell phenotype, blood transcriptomic profile and microbiota identified here in each group may thus serve as a reference for future studies to better understand factors associated with disease severity and ultimately protective adaptive immune responses.”

Reviewer #3:

The authors present results that support their hypothesis that the hygienic and thus the immunological status of a pig determines the course of the disease after ASFV infection. For this purpose, they compare SPF pigs with animals from conventional husbandry. In addition to comparative studies of naïve leucocytes and their transcriptome profiles, the disease courses and immunological reactions after infection with ASFV are described. Here, infections with highly pathogenic virus are compared with those with naturally attenuated ASF virus.
The experimental design is clearly structured. The results are presented logically and very well comprehensible according to the hypothesis and they are discussed extensively in the context of the current literature. They make a valuable contribution to the understanding of the immunopathogenesis of ASF and should be considered in particular for vaccine production based on attenuated viruses.

**Response:**

We thank the reviewer for the positive comments.

**Part II – Major Issues: Key Experiments Required for Acceptance**
Please use this section to detail the key new experiments or modifications of existing experiments that should be absolutely required to validate study conclusions.

Generally, there should be no more than 3 such required experiments or major modifications for a "Major Revision" recommendation. If more than 3 experiments are necessary to validate the study conclusions, then you are encouraged to recommend "Reject".

Reviewer #1:

1) Authors should re-write the paper under the light of previous findings showing the opposite results than those here presented (not even cited).

Together with this main concern, authors should include additional experiments

2) Functional assays are missed (Intracellular cytokine assays, ELISPOs etc…). At least at the time of sacrifice of the surviving pigs….
3) Experiments attempting to address the mechanisms….also missed in here

**Response:**

The manuscript has been re-written to acknowledge previous studies using SPF pigs. In our study, we provide a deep characterization of the basal immune status of the SPF and farm pigs including blood transcriptomic, hematological, immunophenotyping, cytokine response, and microbiota analysis. These parameters provide a range of potential new leads to be further investigated by our group together with the ASFV research community to delineate mechanistic links between health/immune status in response to infection with ASFV genotype II strains of different virulence.

Moreover, we have established that SPF pigs from our facilities have a stable immune and microbiota status over time, which provides a strong model for reproducible analysis of adaptive immune responses that correlates with protection upon rechallenge with a virulent strain. The functional assays suggested by the reviewer are important but beyond the scope of this manuscript. As described above, we are currently performing studies of the adaptive immune responses suggested by the reviewer in the context of a primo infection with the moderately virulent strain Estonia 2014 followed by a challenge with the virulent Armenia 2008 strain. These results require further analysis and replication; they are beyond the scope of the current study.

Reviewer #2: (No Response)

Reviewer #3:

However, I unfortunately have some criticisms concerning the gating strategy of the FACS experiments (Fig. S2 and the resulting figures).
1. The gd T cells are described as CD3+ CD4-CD8-. This is inaccurate. There is indeed a distinct population of CD8+ gd T cells with effector function, which also changes in number in the course of the infection. In the gating used here, these cells are not assigned to the gd T cells, they are found among the CD8+ ab T cells. (In addition, the gate referred to here as gd T cells also contains CD4CD8 double negative ab T cells.) If this cannot be corrected with the existing staining, it should be mentioned and evaluated in the discussion.

**Response:**

We fully agree with the reviewer that labeling CD3+CD4negCD8neg cells as γd T cells is an approximation. We have modified the manuscript accordingly.

Lines 217-221: “This difference was due to reduced numbers of neutrophils, monocytes, and most T cell subsets in SPF pigs, except CD4^-^CD8a^-^ T cells, which are principally composed of γδ T cells and were higher in SPF pigs. Of note, terminally differentiated effector γδ T cells are also present in the CD4^-^CD8a^+^ cytotoxic T cell gate and this specific subset could not be distinguished here from cytotoxic αβ T cells.”

We have also further discussed T cell responses in the context of published studies:

Lines 280-285: “Furthermore, detailed flow cytometry studies of T cell responses to infection with ASFV genotype II strains Estonia 2014 and Armenia 2008 recently showed that severe disease is associated with impaired T cell responses in blood and tissues of domestic pigs and wild boars (Huhr et al., 2020; Schafer et al., 2021). These studies and previous seminal studies provide further impetus to investigate the functions of CD8^+^ (αβ and γδ) T cells and particularly perforin^+^ cells in the pathogenesis and protective immune responses to ASFV (Bosch-Camos et al., 2020; Schafer et al., 2022; Takamatsu et al., 2013).”

2. The use of the anti CD45 antibody seems incomprehensible: In Fig. S2 A and B it is used to clearly identify leukocytes. Why are CD45+ and CD45- CD3- cells used to identify NK cells in Fig. C and D? Different amounts of non-leukocytes in the individual pigs could influence the results.

**Response:**

We apologize for the confusion. We have modified the Figure S2 to clarify the gating of lymphoid cells and to avoid confusion.

As showed for the myeloid cells (panels A and B), lymphoid cells (panels C and D) were also first gated using CD45 and side scatter after doublet exclusion. The new figure now shows this initial step that was of course common for myeloid and lymphoid panels. We hope that showing again the first step will now be clear for our readers.

In the NK gating, the dot plot shows two populations of CD3neg cells: one subset is CD45high and the other shows lower CD45 staining. The cells that appear CD3negCD45low in panels C and D are in fact the SSChi cells, which express lower levels of CD45. We hope that this clarifies the confusion and we thank the reviewer for this comment as we were able to improve the figure layout.

The total amounts of leukocytes (WBC) determined using the hematology counter is set to correspond to the same gate as CD45+ cells for both myeloid and lymphoid panels.

**Part III – Minor Issues: Editorial and Data Presentation Modifications**
Please use this section for editorial suggestions as well as relatively minor modifications of existing data that would enhance clarity.

Reviewer #1:

Any idea of why SPF pigs show fewer monocytes in the blood than conventional pigs? Does the same thing happen in tissues? This observation might be important since macrophages are the main ASFV target and their proportion and number might influence the infection kinetics. This issue should be better addressed in the discussion section

**Response:**

These are indeed interesting questions, but the experimental design did not permit to study organs at different time points during infection. We have added a discussion of these points to the manuscript.

Lines 221-226: “We did not investigate if target cells of ASFV such as macrophages and dendritic cells, which are in part derived from circulating monocytes and in part derived from fetal precursors are also reduced in tissues of SPF pigs before and during infection. However, the faster appearance of viremia in SPF pigs compared to farm pigs inoculated with either virus suggests that a potential lack of target cell numbers in SPF pigs is not a likely factor influencing the initial viral replication kinetics of ASFV *in vivo*.“

Reviewer #2: Some discussion of the early viremia in the Armenia infected pigs is warranted. These and related viruses have been characterized extensively using different infection routes and viremia is typically seen 3 dpi, as opposed to 1 dpi reported here. The dose used by the authors is also relatively low.

**Response:**

Indeed, the viremia was detected faster in SPF pigs (1 dpi) compared to farm pigs (2 dpi), however genome equivalents reported were low at these time points. A higher sensitivity of the qPCR method may explain the early detection. Furthermore, the intramuscular inoculation leads to a faster onset of the viremia. In our hands, direct comparison of infection of SPF pigs with a similar Georgia 2007 strain via the oro-nasal or the intramuscular route led to an earlier detection (day 1) for those inoculated intramuscularly compared to the oronasal route.

Reviewer #3: Minor points also Fig S2:
The neutophils are gated as CD45+SSChi CD14+. Consequently, they should also be named as such in the legend.
The axis labels with CD4a should only be called CD4.

**Response:**

Thank you. We have changed the legend and figure accordingly.

PLOS authors have the option to publish the peer review history of their article ([what does this mean?](https://journals.plos.org/plospathogens/s/editorial-and-peer-review-process#loc-peer-review-history)). If published, this will include your full peer review and any attached files.


If you choose “no”, your identity will remain anonymous but your review may still be made public.


**Do you want your identity to be public for this peer review?** For information about this choice, including consent withdrawal, please see our [Privacy Policy](https://www.plos.org/privacy-policy).

Reviewer #1: No

Reviewer #2: No

Reviewer #3: No

Figure Files:

While revising your submission, please upload your figure files to the Preflight Analysis and Conversion Engine (PACE) digital diagnostic tool, [https://pacev2.apexcovantage.com](https://pacev2.apexcovantage.com/). PACE helps ensure that figures meet PLOS requirements. To use PACE, you must first register as a user. Then, login and navigate to the UPLOAD tab, where you will find detailed instructions on how to use the tool. If you encounter any issues or have any questions when using PACE, please email us at [figures@plos.org](mailto:figures@plos.org).

Data Requirements:

Please note that, as a condition of publication, PLOS' data policy requires that you make available all data used to draw the conclusions outlined in your manuscript. Data must be deposited in an appropriate repository, included within the body of the manuscript, or uploaded as supporting information. This includes all numerical values that were used to generate graphs, histograms etc.. For an example see here on PLOS Biology: <http://www.plosbiology.org/article/info%3Adoi%2F10.1371%2Fjournal.pbio.1001908#s5>.

Reproducibility:

To enhance the reproducibility of your results, we recommend that you deposit your laboratory protocols in [protocols.io](http://protocols.io), where a protocol can be assigned its own identifier (DOI) such that it can be cited independently in the future. Additionally, PLOS ONE offers an option to publish peer-reviewed clinical study protocols. Read more information on sharing protocols at <https://plos.org/protocols?utm_medium=editorial-email&utm_source=authorletters&utm_campaign=protocols>
